# A H_2_S-Nampt Dependent Energetic Circuit Is Critical to Survival and Cytoprotection from Damage in Cancer Cells

**DOI:** 10.1371/journal.pone.0108537

**Published:** 2014-09-23

**Authors:** Reiko Sanokawa-Akakura, Elena A. Ostrakhovitch, Shin Akakura, Scott Goodwin, Siamak Tabibzadeh

**Affiliations:** 1 Frontiers in Bioscience Research Institute in Aging and Cancer, Irvine, California, United States of America; 2 Dept of Radiological Sciences, University of California Irvine, Irvine, California, United States of America; University of Iowa, United States of America

## Abstract

We recently demonstrated that cancer cells that recover from damage exhibit increased aerobic glycolysis, however, the molecular mechanism by which cancer cells survive the damage and show increased aerobic glycolysis remains unknown. Here, we demonstrate that diverse cancer cells that survive hypoxic or oxidative damage show rapid cell proliferation, and develop tolerance to damage associated with increased production of hydrogen sulfide (H_2_S) which drives up-regulation of nicotinamide phosphoribosyltransferase (Nampt). Consistent with existence of a H_2_S-Nampt energetic circuit, in damage recovered cancer cells, H_2_S, Nampt and ATP production exhibit a significant correlation. Moreover, the treatment of cancer cells with H_2_S donor, NaHS, coordinately increases Nampt and ATP levels, and protects cells from drug induced damage. Inhibition of cystathionine beta synthase (CBS) or cystathionase (CTH), enzymes which drive generation of H_2_S, decreases Nampt production while suppression of Nampt pathway by FK866, decreases H_2_S and ATP levels. Damage recovered cells isolated from tumors grown subcutaneously in athymic mice also show increased production of H_2_S, Nampt and ATP levels, associated with increased glycolysis and rapid proliferation. Together, these data show that upon recovery from potential lethal damage, H_2_S-Nampt directs energy expenditure and aerobic glycolysis in cancer cells, leads to their exponential growth, and causes a high degree of tolerance to damage. Identification of H_2_S-Nampt as a pathway responsible for induction of damage tolerance in cancer cells may underlie resistance to therapy and offers the opportunity to target this pathway as a means in treatment of cancer.

## Introduction

In recent years, it has been shown that gasotransmitters, nitric oxide (NO), carbon monoxide (CO) and hydrogen sulfide (H_2_S), play a critical role in diverse physiological functions, including vascular tone, host defense against pathogens, neuromodulation, apoptosis, and energy metabolism in mammalian cells [1]. Among these gaseous molecules, H_2_S is produced during amino acid metabolism by the trans-sulfuration and cysteine desulfuration pathways [2]. Endogenous H_2_S production is catalyzed by three enzymes, cystathionine beta synthase (CBS), cystathionase (CTH) also known as cystathionine gamma-lyase, and 3-mercaptopyruvate sulfurtransferase (MST) [3]–[5].

H_2_S functions as a stimulator of cellular bioenergetics, contributes to the increased reliance of cancer cells on the glycolytic pathway for ATP production and promotes angiogenesis and cytoprotection [6]–[8]. H_2_S protects cells from oxidative stress [9], it can affect cellular responses to injury [10], [11] and was shown to exhibit both pro-apoptotic and anti-apoptotic effects [12]–[14]. Although some suggested that H_2_S has anti-cancer effects [15], others reported that it promotes proliferation of HCT116 and SW480 colonic cancer cells [16]. Fu *et al.* showed that Ca^2+^ stimulation causes increased CTH expression and increases H_2_S and ATP production [17]. It was shown that endogenous H_2_S production driven by 3-MST complements and balances the cellular bioenergetics and maintains electron flow in mitochondria [18]. Colonic cancer cells have been shown to exhibit up-regulated expression of CBS and increased formation of H_2_S, which directs cell proliferation and angiogenesis in colon cancer [6].

It is known that tumor cells can recover from potential lethal damage induced by hypoxia, acidosis, or by radiation and drug treatment [19]–[22]. We recently reported that cancer cells that recover from damages induced by hypoxia, acidosis and glucose deprivation show mitochondrial remodeling, increased aerobic glycolysis, and exhibit a high rate of ATP production [23]. In this study, we explore the role of H_2_S in the process of recovery of cancer cells from damage. Damaged cancer cells exhaust their energy supply due to repair mechanisms. Both ATP and NAD^+^ (Nicotinamide adenine dinucleotide) are the main energy sources. Nicotinamide phosphoribosyltransferase (Nampt), an enzyme required for NAD synthetic salvage pathway [24], is vital to the maintenance of cellular energy supply. Therefore, we examined the role of Nampt in conjunction with H_2_S in cancer cells that recover from damage. We demonstrate that H_2_S controls the recovery of cancer cells from damage by regulating Nampt directed change in energy expenditure, which drives adoption of aerobic glycolysis and increase in ATP and NAD^+^ synthesis. The interaction of H_2_S and Nampt confers the cancer cells a high proliferation rate and a high degree of tolerance to damage.

## Materials and Methods

### Materials

H_2_O_2_, NaHS, bleomycin, *O*-(carboxymethyl) hydroxylamine hemihydrochloride (CHH) DL-propargylglycine (PAG) and FK866 were purchased from Sigma-Aldrich (St. Louis, MO). Antibodies against CBS (A-2), CTH (G-1) and β-Actin (C-2) were purchased from Santa Cruz Biotechnology (Santa Cruz, CA). Antibodies against Nampt (Visfatin, PBEF) and Bax were purchased from Abcam (Cambridge, MA). Antibody against γ-H2AX was purchased from Millipore (Billerica, MA). Secondary antibodies conjugated to horseradish peroxidase were purchased from Jackson ImmunoResearch Laboratories (Baltimore, PA).

### Cell culture

HepG2, MDA-MB-231 and MDA-MB-435S were obtained from ATCC (Manassas, VA) cultured in DMEM with 2 mM glutamine, 25 mM glucose and 10% fetal bovine serum, in 37°C incubator with 5% CO_2_. Damage was induced in cancer cells by hypoxia, glucose deprivation and hydrogen peroxide. For induction of hypoxia (DR^H^), cells were incubated for 18 hr in 1% O_2_. For glucose deprivation (DR^G−^), cells were cultured for 18 hr in a medium without glucose in presence of 5% CO_2_. For induction of damage by hydrogen peroxide (DR^H2O2^), cells were treated with 800 µM H_2_O_2_ for 3 hr.

### Animal experiments

Animal care and all procedures were carried out following approval of the Institutional Animal Care Committees of University of California, Irvine (protocol #2012-3042). Eight-week-old athymic nude (Nu/Nu) male mice (n = 10) were purchased from Charles River Laboratories (San Diego, CA). Mice were anesthetized by inhalation of Isoflurane before injection of tumor cells. Each mouse received 1×10^5^ tumor cells in a total volume of 100 µl subcutaneously in four locations in the mid-abdominal and lower flank areas. Mice were monitored 2 times per week during tumor growth experiment. Twenty days after injection of cells, mice were euthanized by carbon dioxide inhalation when tumors grew to the size of 10–15 mm in diameter. Tumors were removed for isolation of viable cancer cells (T^V^) or damage recovered cells from *in vivo* grown tumor (T^DR^).

### Measurement of H_2_S production in extra and intra-cells

Measurement of extracellular H_2_S level was performed using Free Radical Analyzer (TBR4100 and ISO-H2S-2, World Precision Instruments, Sarasota, FL) following manufacturer's instruction. Briefly, cell number was adjusted to 1×10^6^ viable cells in PBS and the cell suspensions were incubated at 37°C for 1 hr. Cells were then centrifuged and the supernatants were subjected to measurements. Prior to each measurement, the sensor was polarized and calibrated by adding four aliquots of the Na_2_S stock solution at the final concentrations of 0.25, 0.5, 1.0 and 2.0 µM. Detection of intracellular H_2_S was performed by H_2_S fluorescent probe HSN2 (a kind gift from Professor Michael D. Pluth, University of Oregon, Department of Chemistry, Eugene, Oregon).

### Whole cell protein extraction and Western blotting

Proteins from cells were extracted in lysis buffer (50 mM Tris-HCl, pH 7.5, 150 mM NaCl, 1% NP-40, 2 mM EDTA, 1 mM PMSF, 1 mM Na_3_VO_4_, 50 mM NaF, and protease inhibitor cocktail). Protein measurements were carried out by Bio-Rad protein assay based on Bradford dye-binding method (Bio-Rad Lab, Hercules, CA).

Blotting bands were detected by ECL enhanced chemiluminescence (Amersham ECL Plus Western Blotting Detection Reagents GE Healthcare Life Sciences, Pittsburgh, PA) using C-Digit Digital Imager (LI-COR, Lincoln, NE) and densitometric analysis was performed using myImage Analysis software (Thermo Scientific). β-actin served as a loading control.

### Cell viability measurement

Relative cell number was measured by XTT assay (Sigma-Aldrich, St. Louis, MO). Cells were incubated with XTT and phenazine methosulfate (PMS) at 37°C for 2 hr and absorbance was read at 450 and 650 nm as a reference.

### Reverse transcription-Polymerase chain reaction (PCR) and Quantitative PCR (qPCR)

Total RNA was isolated using GenElute Mammalian Total RNA Miniprep Kit (Siγma-Aldrich, St. Louis, MO). Reverse transcription was performed using High-Capacity cDNA Reverse Transcription Kit (Applied Biosystems, Carlsbad, CA). RT-PCR was carried out using the primers specific for the human CBS (forward: GAACCAGACGGAGCAGACAA; reverse: GTCGCTCAGGAACTTGGTCA), for the human CTH (forward: AAAGACGCCTCCTCACAAGG; reverse: AAGGCAATTCCTAGTGGGATTTC) and for the human MTS (forward: CGCCGTGTCACTGCTTGAT; reverse: CAGGTTCAATGCCGTCTCG). Gene expression was assessed by PCR using *Taq* 5× Master Mix (New England Biolabs. Ipswich, MA) with an initial denaturation step 94°C for 5 min, followed by 30 cycles with each at 94°C for 30 sec, 55°C for 30 sec, and 68°C for 1 min.

Quantitative evaluation was performed by using myImage Analysis software (Thermo Scientific, New Hampshire). For normalization of data, *β-ACTIN* was amplified with specific primers (forward: AAGCCACCCCACTTCTCTCT; reverse: GAGACCAAAAGCCTTCATACATCT). qPCR was performed using Quanti Tect SYBR Green PCR Kit. qPCR was carried out using the primers specific for the human NAMPT (forward: ATC CTG TTC CAG GCT ATT CTG; reverse: CCC CAT ATT TTC TCA CAC GCA T).

### XF (extracellular flux) bioenergetic analysis

XF24 Extracellular Flux Analyzer from Seahorse Bioscience (N. Billerica, MA) was utilized for extracellular fluid bioenergetic analysis [25]. Under typical *in vitro* cell culture conditions, extracellular acidification rate (ECAR) is contributed by lactic acid production generated by glycolysis. To examine the aerobic glycolysis, five biological replicate cultures of control (3×10^4^) and five independently generated DR cells (3×10^4^) were plated in each well of the XF 24 culture plate and cells were incubated overnight at 37°C in presence of 5% CO_2_. All cultures were examined in XFAssay media in the absence of CO_2_. Level of ECAR was normalized to the protein content.

### Quantitation of Nampt, ATP and NAD^+^/NADH

Intracellular Nampt levels were measured using a Visfatin C-terminal (Human) EIA kit (Phoenix pharmaceuticals, Belmont, CA, USA). ATP levels in cells was assayed using the Bioluminescent ATP Somatic cell assay kit (FLASC, Sigma-Aldrich company) and colorimetric ATP assay kit (Abcam, Cambridge, MA) according to manufacturer's instruction. NAD^+^/NADH levels were measured using NAD^+^/NADH assay kit (Cayman Chemical Company, Ann Arbor, Michigan and Bio Assay Systems, Hayward CA) following manufacturer's instruction. Levels of Nampt, ATP and NAD^+^/NADH were normalized to the protein content.

### Statistics

All assays were done in 3–6 replicates in at least three independent experiments. Data are shown as mean ± SEM (standard error of the mean). *p* values were determined by comparing the data from experimental versus control cells from at least three independent experiments or six replicates of the same experiment. Means and *p* values for experimental data were analyzed by subjecting the data to the two tailed t-test. Data involving more than two groups were accessed by one-way ANOVA with Bonferroni's test for post hoc analysis. *p* values less than 0.05 were considered significant. *p* Values are shown as <0.05 (*), <0.005 (**) or <0.0005 (***).

## Results

### The generation of H_2_S increases in response to stress

**Figure 1:**
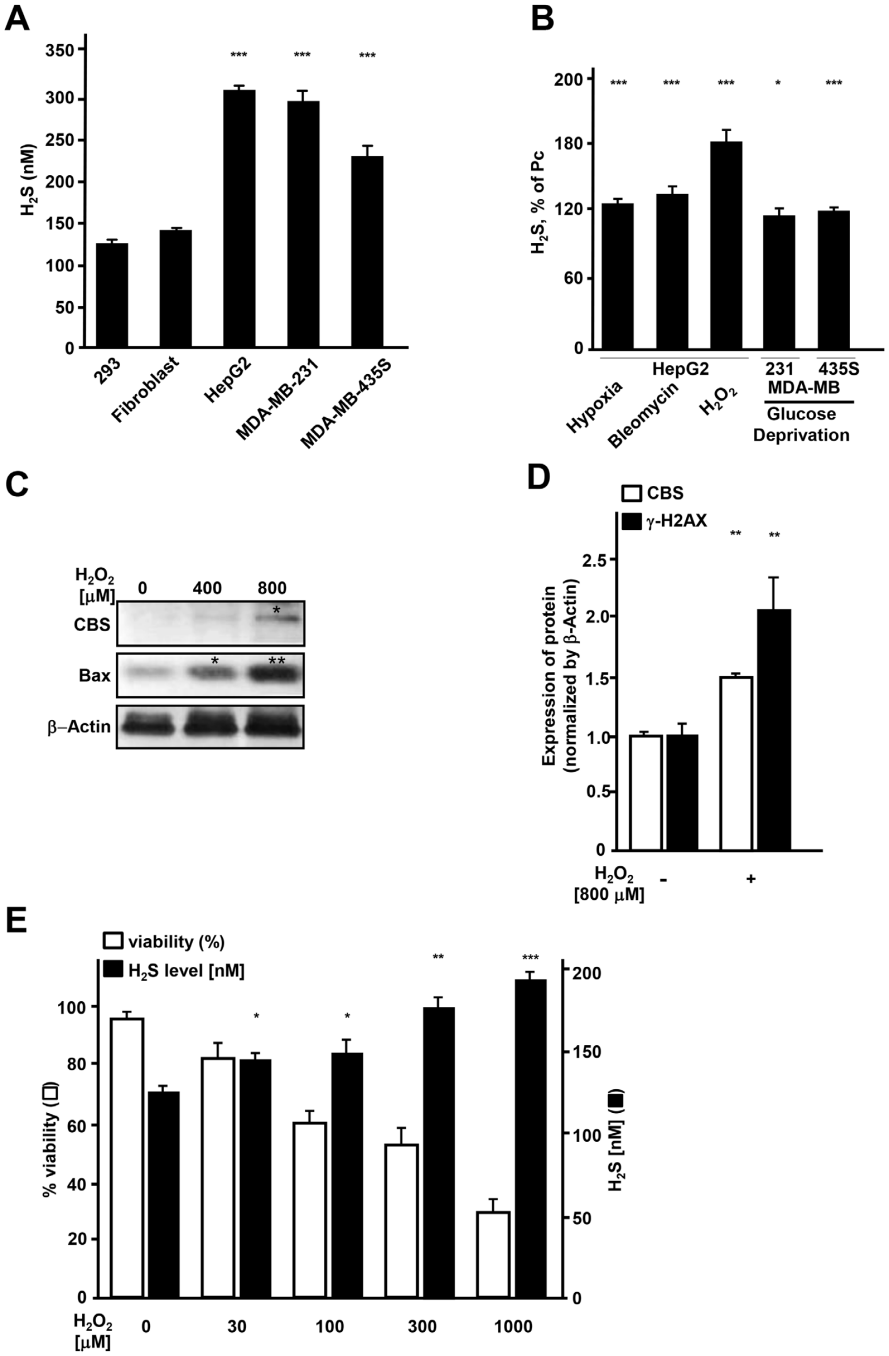
Endogenous hydrogen sulfide increases in response to acute damage in cancer cells. (A) Amount of H_2_S released by 293 cells, fibroblasts (Fibro.), HepG2, MDA-MB-231 and MDA-MB-435S cells. (B) The levels of H_2_S in HepG2, MDA-MB-231 and MDA-MB-435S Pc cells subjected to hypoxia (0.5% O_2_, 18 hr), glucose deprivation (glucose free medium, 18 hr) or treatment with bleomycin (35 nM, 18 hr), H_2_O_2_ (800 µM, 3 hr). Data are expressed as percent of H_2_S released from untreated cells. (C) Intracellular CBS and Bax (left panel) assessed by western blot analysis in MDA-MB-435S Pc cells, and Pc cells treated with 400 or 800 µM of H_2_O_2_ for 3 hr. (D) The level of CBS and γH2AX with or without treatment with H_2_O_2_ (800 µM, 3 hr) in MDA-MB-435S cells. Protein density was normalized using β-Actin. (E) Amount of H_2_S and cell viability after treatment with a range of concentration of H_2_O_2_. *; *p*<0.05,**; *p*<0.005, ***; *p*<0.0005.

In contrast to previous reports in which H_2_S has been measured in cell lysate, we utilized H_2_S-sensitive electrode to measure the release of H_2_S from intact cells. We found that the amount of H_2_S released from cells was significantly higher than that measured in homogenized cell extracts (data not shown). In comparison with the levels of H_2_S released from 293 cells and fibroblasts, cancer cells (HepG2, MDA-MB-231, MDA-MB-435S) produced significantly more H_2_S (Figure 1A). We subjected epithelial cancer cells of different origins (liver, breast, and melanocyte) to harsh conditions that exist in the tumor microenvironment, which all lead to cell damage including hypoxia (^H^), glucose deprivation (^G−^) and hydrogen peroxide (^H2O2^). The H_2_S generation was increased in cancer cells in response to acute stress such as hypoxia, bleomycin, hydrogen peroxide and glucose deprivation (Figure 1B). Stress related rise in H_2_S released from cancer cells was associated with increase in cystathionine beta synthase (CBS), one of the enzymes that drives H_2_S production, concomitant with increase in stress and apoptosis-related Bax and γH2AX, which is a critical factor of the S/G_2_ DNA-damage checkpoint complex (Figure 1C, D). Interestingly, stress induced elevation in H_2_S production correlated with the severity of damage (Figure 1E).

### The increased generation of H_2_S in damage-recovered cells increases tolerance to damage

**Figure 2:**
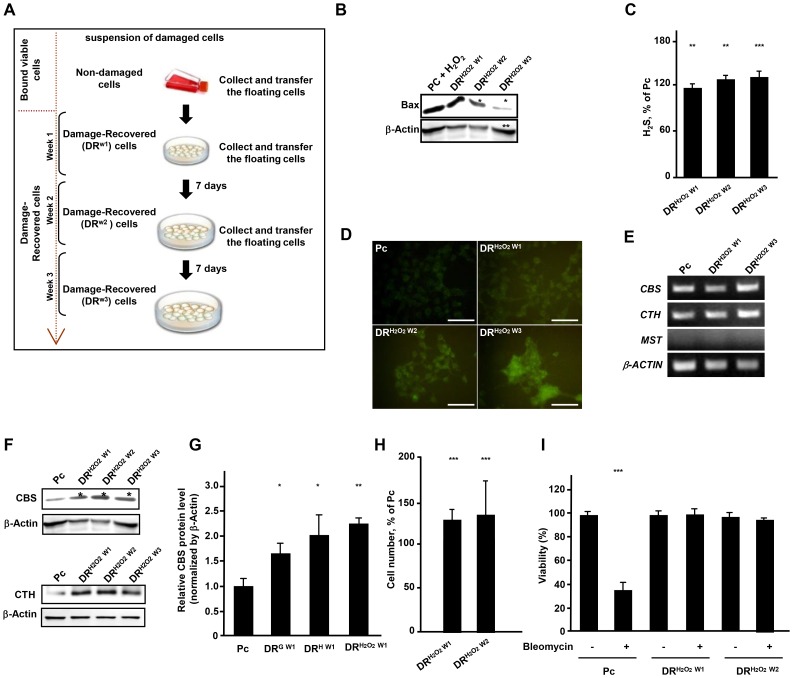
Damage-Recovered (DR) cells show increase in H_2_S and proliferation rate and exhibit tolerance to damage. (A) A scheme for isolation of Damage-Recovered (DR) cells. (B) Bax expression in H_2_O_2_ treated Pc, DR^H2O2 W1^, DR^H2O2 W2^ and DR^H2O2 W3^ HepG2 cells. (C) Amount of H_2_S released by DR^H2O2 W1^, DR^H2O2 W2^ and DR^H2O2 W3^ HepG2 cells. Significance between Pc and three DR cells was *p*<0.0005 in ANOVA statistical analysis. (D) H_2_S staining of Pc, DR^H2O2 W1^, DR^H2O2 W2^ and DR^H2O2 W3^ HepG2 cells with 5 µM H_2_S fluorescent probe, HSN2. Scale bars, 50 µm. (E) PCR analysis of *CBS*, *CTH* and *MTS* genes in Pc, DR^H2O2 W1^ and DR^H2O2 W3^ HepG2 cells. (F) Western blot analysis of CBS and CTH in Pc, DR^H2O2 W1^, DR^H2O2 W2^ and DR^H2O2 W3^ HepG2 cells. (G) Western blot analysis of CBS in Pc and DR^H2O2 W1^, DR^H W1^ and DR^G W1^ HepG2 cells. (H) Proliferation of HepG2 recovered from H_2_O_2_, DR^H2O2 W1^, DR^H2O2 W2^ cells as a percentage of that in Pc cells. (I) Viability of Pc, DR^H2O2 W1^ and DR^H2O2 W2^ HepG2 cells with and without treatment with bleomycin. *; *p*<0.05,**; *p*<0.005, ***; *p*<0.0005.

The scheme in Figure 2A shows the method that we used to generate the damage recovered cancer cells. Within 24 hr after damage, few viable cells remained attached to the culture flask while majority of cells detached from culture surface. Damaged detached cells exhibit a high level of H_2_S likely to limit the injury and can improve survival potential (Figure S1). To remove the mitotic cells, we cultured cells in new culture vessels for 24 hr. To isolate damaged cells that failed to bind to culture vessels but had the potential to recover from damage, we transferred floating cells to new culture vessels to allow cells that recover from damage to bind to the culture substrates. Serial weekly passages of floating cells to new culture vessels allowed isolation of damage recovered cells with different recovery time. Throughout this paper, we refer to these cells as damage-recovered (DR) cells with indicated recovery time of one (DR^W1^), two (DR^W2^) or three weeks (DR^W3^) from damage, while we refer to the parental control cells as Pc cells (Figure 2A). This method enabled us to separate three populations of cells that reflect the length of time required for recovery. In order to assess whether isolated DR cells recovered from damage, we examined the expression of pro-apoptotic molecule, Bax. DR cells showed a decrease in Bax expression in a recovery time dependent manner as an indication of repair, while as predicted, Pc cells exposed to H_2_O_2_ for 3 hr (acute damage) expressed a high level of Bax (Figure 2B). Furthermore, cancer cells recovered from damage also exhibited an increased production of H_2_S as compared to the undamaged Pc cells in a recovery time dependent manner (Figure 2C, D). Since endogenous hydrogen sulfide is generated by three enzymes, CBS, CTH and MST, we examined mRNA and protein levels of these enzymes in Pc and DR cells. As shown in Figure 2E, mRNA levels of CBS and CTH increased in DR cells also in a recovery time dependent manner. However, MST was absent in Pc or DR cells. As compared to parental undamaged Pc cells, cancer cells recovered from damage had increased CBS and CTH proteins in direct correlation with the recovery period. Among the DR cells, those cells with a longer recovery time from damage show the highest up-regulation of CBS and CTH (Figure 2F). CBS was up-regulated up to 2 fold after recovery from damage induced by glucose deprivation, hypoxia and hydrogen peroxide (Figure 2G). As compared to Pc cells, DR cells generating higher levels of H_2_S had a higher proliferation rate and showed a higher level of tolerance to damage induced by bleomycin (Figure 2H, I). These findings show that in cells recovered from damage, H_2_S production might play a role in their increased tolerance to damage.

### DR cells exhibit increased glycolysis and enhanced cellular bioenergetics

**Figure 3:**
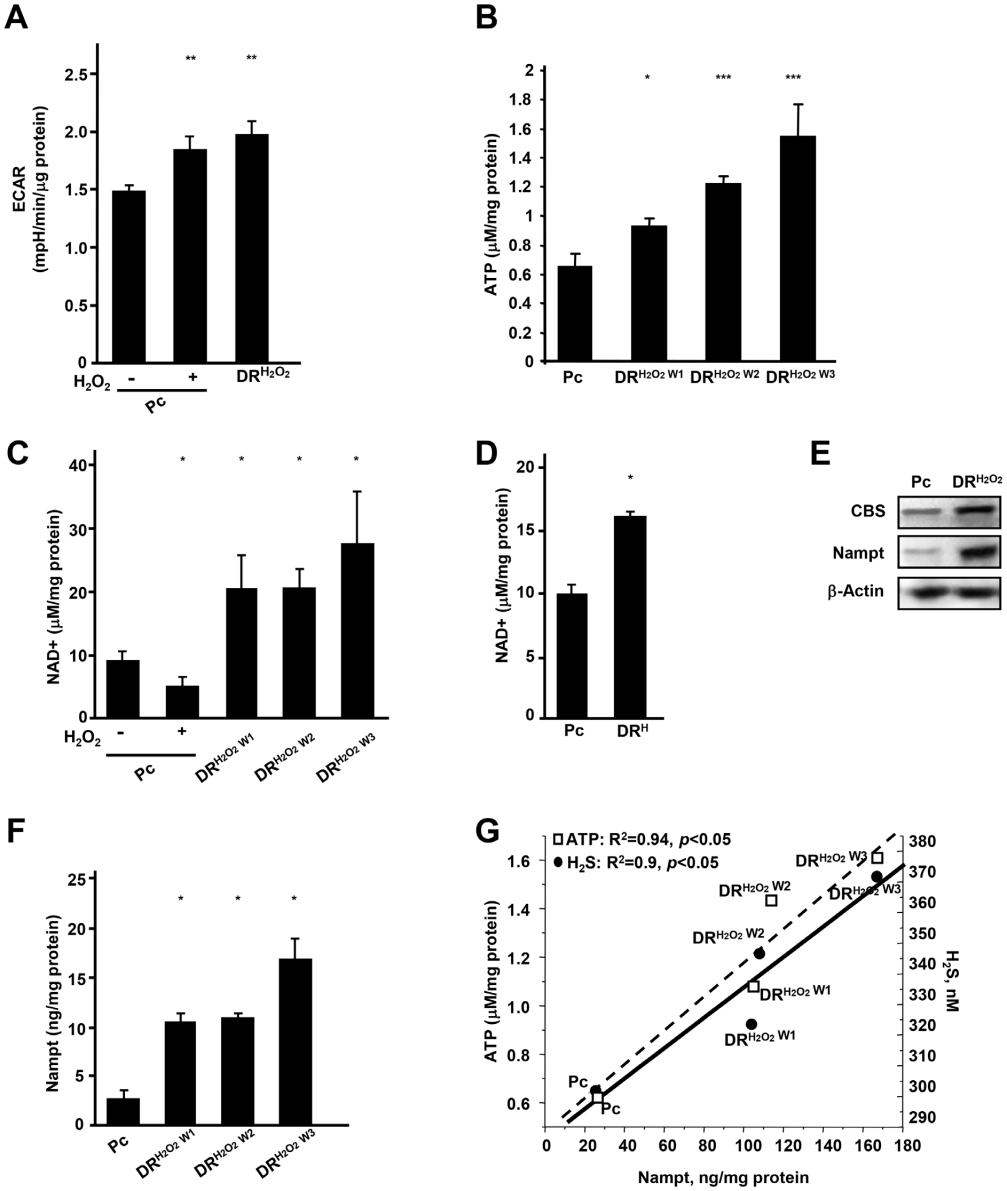
Changes in glycolysis and bioenergetics in DR cells. (A) ECAR in Pc HepG2 cells treated with or without 800 µM of H_2_O_2_ and DR^H2O2 W2^ HepG2 cells. (B) ATP levels in Pc, DR^H2O2 W1^, DR^H2O2 W2^ and DR^H2O2 W3^ HepG2 cells. Significance between Pc and three DR cells was *p*<0.005 in ANOVA statistical analysis. (C) NAD^+^ levels in Pc HepG2 (with or without H_2_O_2_), DR^H2O2 W1^, DR^H2O2 W2^ and DR^H2O2 W3^ HepG2 cells. NAD^+^ was normalized to the level of total protein. Significance between Pc and there DR cells was *p*<0.005 in ANOVA statistical analysis. (D) NAD^+^ levels in Pc and DR^H W1^ HepG2 cells. (E) Western blot analysis of intracellular Nampt and CBS in Pc and DR^H2O2^ MDA-MB-231 cells. (F) Nampt levels assessed by ELISA in Pc, DR^H2O2 W1^, DR^H2O2 W2^ and DR^H2O2 W3^ HepG2 cells. Significance between Pc and three DR cells was *p*<0.05 in ANOVA statistical analysis. (G) Correlation between Nampt expression and production of H_2_S and level of ATP in Pc, DR^H2O2 W1^, DR^H2O2 W2^ and DR^H2O2 W3^ HepG2 cells. Significance of H_2_S and Nampt was *p*<0.05, and significance of ATP and Nampt was *p*<0.05 in ANOVA statistical analysis. *; *p*<0.05,**; *p*<0.005, ***; *p*<0.0005.

Because rapidly dividing cells adopt aerobic glycolysis to promote an increased biomass followed by cell division, we examined the level of key glycolytic intermediates and enzymes in cells recovered from damage. DR cells showed an elevated extracellular acidification rate (ECAR), which is an indicator of level of glycolysis (Figure 3A). DR cells generating high levels of H_2_S had a better bioenergetic profile as evidenced by increased level of cellular ATP (Figure 3B).

In order to address whether suppression of glycolysis attenuates H_2_S production, we measured H_2_S level in DR cells treated with HK1 inhibitor, 100 µM bromopyruvic acid, or LDH-A inhibitor, 1 mM sodium oxamate, for 15 hr. As shown in Figure S2, there was no statistically significant difference in the level of H_2_S after treatment with either bromopyruvic acid or sodium oxamate, whereas, as expected, glycolytic activity was substantially diminished by both inhibitors. These data indicate that glycolytic enzymes do not regulate H_2_S-Nampt circuit.

Cells recovered from damage heavily relied on glycolysis that increased demand for NADH/NAD^+^. Therefore, as a measure of the bioenergetic state, we evaluated the levels of NAD^+^ and NADH. Although acute damage led to a decrease in the level of NAD^+^, NAD^+^ was significantly increased in cancer cells that recovered from damage induced by H_2_O_2_ and hypoxia (Figure 3C, D; Figure S3). Reduced form of NAD^+^, NADH, was significantly increased in DR cells (Figure S4).

Nampt, which is required for NAD^+^ synthetic salvage pathway [24], was significantly increased upon recovery in DR cells and this increase coincided with up-regulation of H_2_S generating CBS (Figure 3E). The DR cells with longer recovery time from damage showed the highest increase in Nampt (Figure 3F). Moreover, we found that Nampt levels correlated with both the level of H_2_S released from cells (R^2^ = 0.9, *p*<0.05) and the intracellular level of ATP (R^2^ = 0.94, *p*<0.05) (Figure 3G).

Together, these findings show that cancer cells recovered from damage generate a high level of H_2_S and Nampt, and that they are heavily dependent on the production of H_2_S and aerobic glycolysis for their efficient metabolism.

### H_2_S, in a dose-dependent manner, up-regulates Nampt, increases aerobic glycolysis and affords cytoprotection

**Figure 4:**
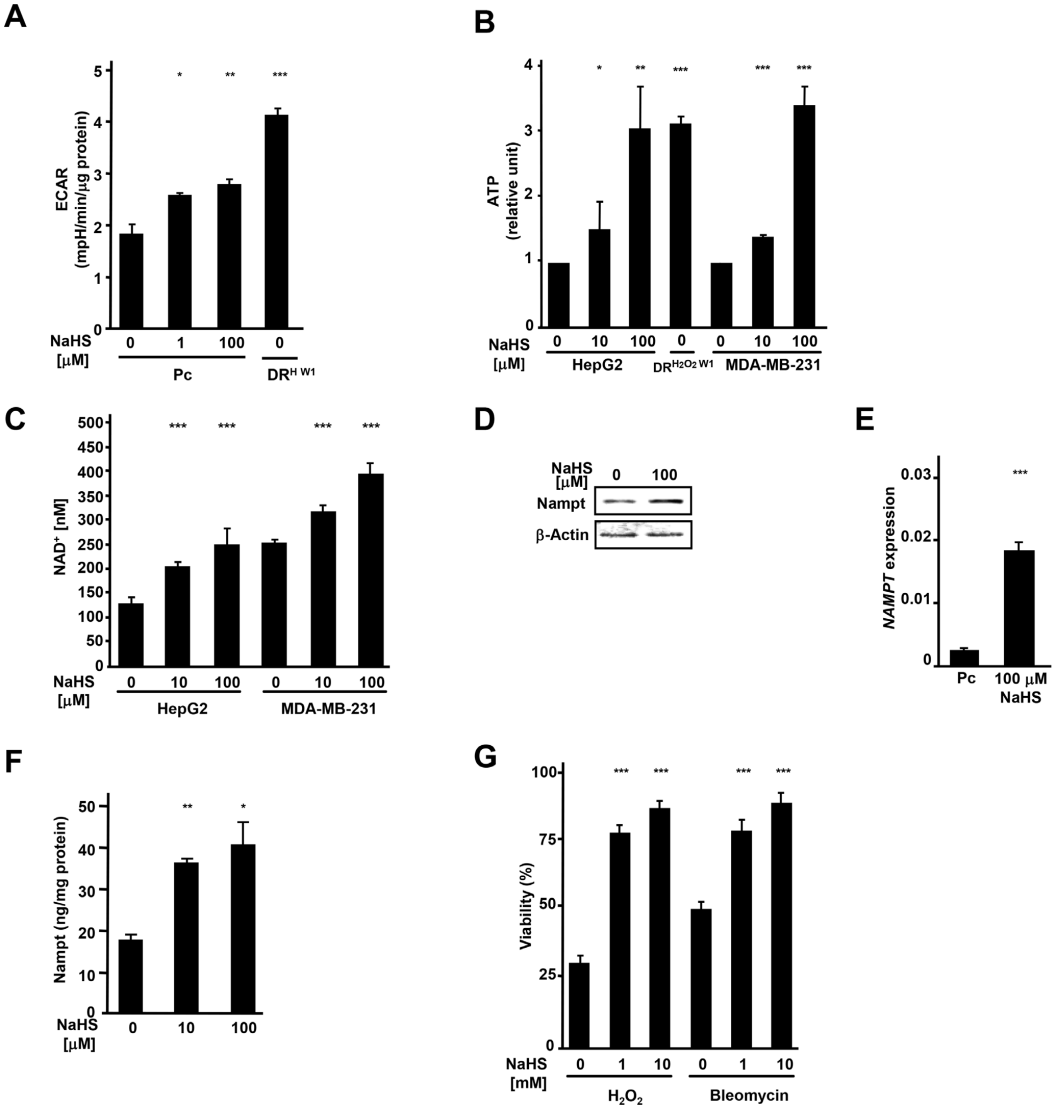
H_2_S increases ECAR, ATP, NAD^+^ and Nampt in a dose-dependent manner in cancer cells. (A) ECAR in Pc HepG2 cells treated for 48 hr with 0, 1, and 100 µM of NaHS and DR^H W1^ HepG2 cells. (B) Comparison of ATP levels in Pc HepG2 cells treated for 48 hr with 0, 10 and 100 µM of NaHS, DR^H2O2 W1^ HepG2 cells and Pc MDA-MB-231 cells treated for 48 hr with 0, 10 and 100 µM of NaHS. Data are expressed as a percent of level of ATP in untreated cells. (C) Levels of NAD^+^ in HepG2 and MDA-MB-231 Pc cells treated with 0, 10, and 100 µM of NaHS for 48 hr. (D) Western blot analysis of intracellular Nampt in MDA-MB-231 Pc cells treated for 48 hr with 0 and 100 µM of NaHS. (E) qPCR analysis of *NAMPT* expression in Pc HepG2 cells treated for 48 hr with 0 and 100 µM of NaHS. (F) Quantitation of intracellular Nampt by ELISA in Pc HepG2 cells treated for 48 hr with 0, 10 and 100 µM of NaHS. (G) Viability in HepG2 cells pre-treated for 24 hr with 0, 1, and 10 mM of NaHS and then subjected to H_2_O_2_ (800 µM) or bleomycin (35 nM, 18 hr). Viability was assessed by Trypan blue exclusion. *; *p*<0.05,**; *p*<0.005, ***; *p*<0.0005.

To further assess the importance of H_2_S in acquisition of new metabolic characteristics, Pc cells were treated with H_2_S donor, NaHS. Similar to our observations in DR cells (Figure 3A), treatment with NaHS enhanced ECAR in a dose dependent manner, increased ATP and NAD^+^ output and led to significant rise in the level of Nampt (Figure 4A–F). Consistent with previous reports showing that H_2_S provides cytoprotection [8], [26], Pc cells pre-treated with NaHS exhibited a higher resistance to damage induced by either hydrogen peroxide or bleomycin (Figure 4G).

Together, our findings show that up-regulation of H_2_S and Nampt leading to glycolysis and bioenergetic changes are important mechanisms in the development of drug resistance and survival of cancer cells.

### H_2_S-Nampt pathway regulates bioenergetics in DR cells

**Figure 5:**
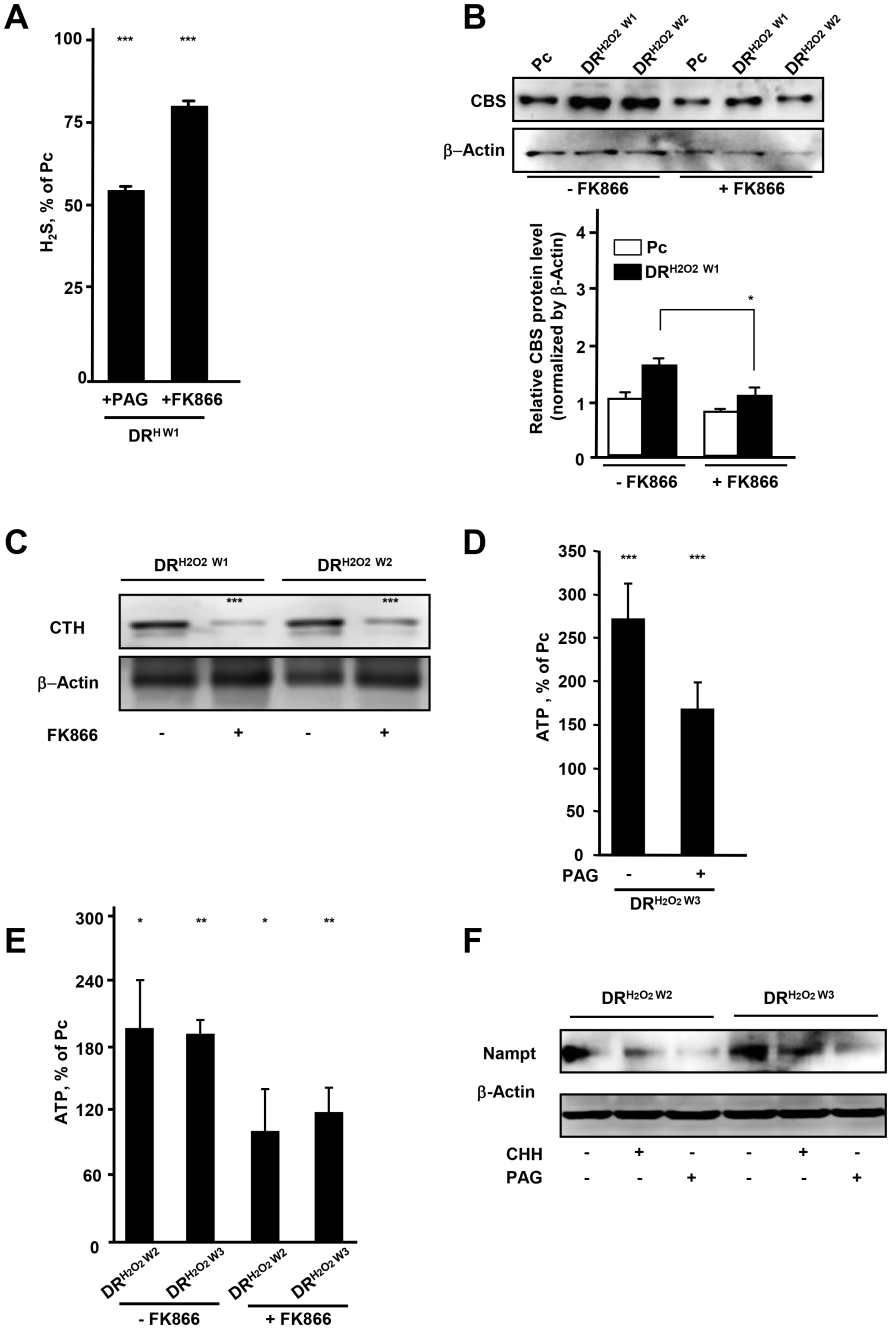
H_2_S-Nampt pathway regulates bioenergetics. (A) Amount of H_2_S released from DR^H W1^ HepG2 cells treated with CTH inhibitor, PAG (100 µM, 18 hr), and Nampt inhibitor, FK866 (200 nM, 24 hr). (B) Western blot analysis of CBS in DR cells in the absence and presence of FK866 (200 nM, 24 hr). (C) Western blot analysis of CTH in DR cells in the absence and presence of FK866 (200 nM, 24 hr). (D) ATP levels in DR^H2O2 W3^ HepG2 cells in the absence (−) and presence (+) of PAG (100 µM, 18 hr). (E) ATP levels in Pc, DR^H2O2 W2^ and DR^H2O2 W3^ HepG2 cells in the absence and presence of FK866 (200 nM, 24 hr). (F) Western blot analysis of Nampt in DR^H2O2 W2^ and DR^H2O2 W3^ HepG2 cells treated with CBS inhibitor, CHH (500 µM, 18 hr) or CTH inhibitor, PAG (100 µM, 18 hr). *; *p*<0.05,**; *p*<0.005, ***; *p*<0.0005.

To further address the relation between Nampt and H_2_S production, DR cells were treated with an inhibitor of Nampt, FK866. Treatment of cells with 200 nM of FK866 did not affect cell viability indicating the absence of FK866 cytotoxicity at this dose in HepG2 (Figure S5). Suppression of Nampt by its inhibitor, FK866, as well as inhibition of CTH by D, L-propargylglycine (PAG), led to decreased H_2_S production (Figure 5A). Consistent with such a change in H_2_S production, the expression of both CBS and CTH was attenuated by treatment of cells with FK866 (Figure 5B, C). The level of ATP was also significantly decreased by treatment of DR cells with PAG and FK866 (Figure 5D, E). This reduction of ATP was consistent with previous study showing that inhibition of Nampt by FK866 suppressed the production of ATP in ovarian cancer cells [27]. Interestingly, treatment of DR cells with inhibitor of CBS (CHH) and inhibitor of CTH (PAG) reduced Nampt (Figure 5F). These data suggest that Nampt may function as a stimulator of H_2_S-producting enzymes and thereby production of H_2_S, whereas H_2_S causes up-regulation of Nampt.

Taken together, our findings show that up-regulation of H_2_S and Nampt and their crosstalk improve bioenergetic efficiency and facilitate recovery from damage.

### Accumulation of H_2_S leads to higher glycolysis, better bioenergetics and increased proliferation in damage recovered cancer cells isolated from *in vivo* grown tumors

**Figure 6:**
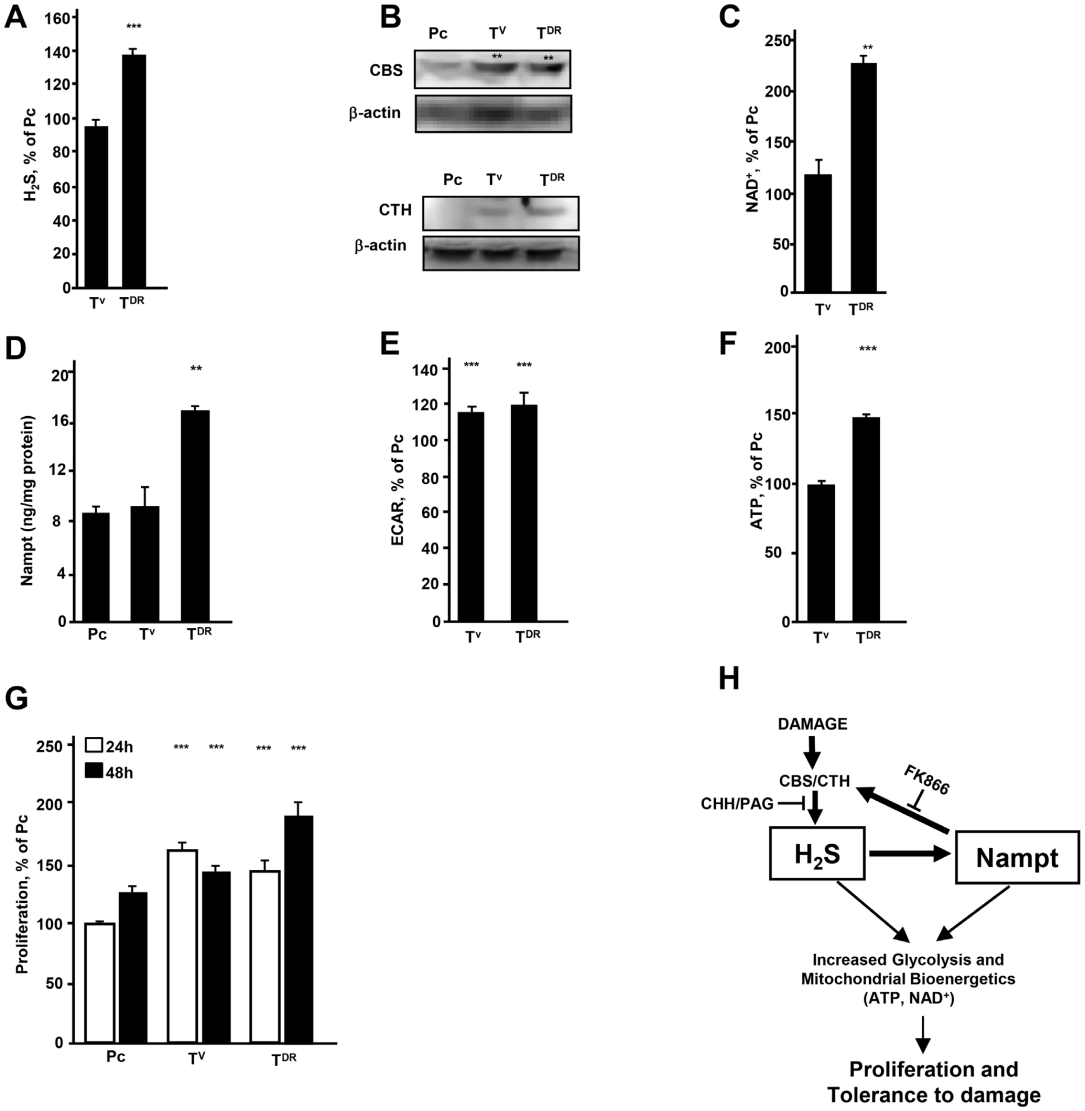
H_2_S levels and bioenergetic changes in DR cells isolated from tumors. (A) H_2_S levels in T^V^ and T^DR^ cells isolated from HepG2 tumors grown *in vivo*. (B) Expression of CBS and CTH in T^V^ and T^DR^ cells. (C) NAD^+^ levels in T^V^ and T^DR^ cells isolated from HepG2 tumors grown *in vivo*. (D) Nampt levels in T^V^ and T^DR^ cells isolated from HepG2 tumors grown *in vivo*. (E) ECAR in T^V^ and T^DR^ cells isolated from HepG2 tumors grown *in vivo* expressed as the percent of the ECAR level in Pc. (F) ATP level in viable tumor cells (T^V^) isolated from HepG2 tumors grown *in vivo* expressed as the percent of the ATP level in Pc. (G) Proliferation of T^V^ and T^DR^ cells isolated from HepG2 tumors grown *in vivo* expressed as the percent of the proliferation of Pc. Cells were seeded at a concentration of 2×10^4^ and the total number of cells was assessed after 24 and 48 hr of culture. (H) A scheme for H_2_S-Nampt dependent bioenergetic circuit. Cell damage leads to increased H_2_S and Nampt that coordinately lead to metabolic changes. These cells exhibit exponential growth and tolerance to damage. *; *p*<0.05,**; *p*<0.005, ***; *p*<0.0005.

To identify whether cells similar to *in vitro* generated DR cells exist or can be generated in tumors, epithelial cancer cells were inoculated into athymic nude mice. We isolated viable cancer cells (T^V^) and damage recovered (T^DR^) cells, as described in our previous study [23]. T^DR^ cells isolated from *in vivo* generated tumors showed the accumulation of H_2_S as well as increased expression of CBS and CTH (Figure 6A, B). Similar to *in vitro* data, the levels of NAD^+^ and Nampt were increased in T^DR^ cells as compared to the levels detected in Pc and T^V^ cells (Figure 6C, D). ECAR was increased in T^DR^ cells and these cells showed a better bioenergetic profile as evidenced by increased level of ATP and a higher proliferation rate (Figure 6E–G).

Based on these findings, we propose a scheme whereby cancer cells recovered from damage change their metabolic profile via up-regulation of H_2_S-Nampt pathway (Figure 6H). Thus, H_2_S-Nampt dependent energetic circuit is a critical regulator of stress tolerance, increased glycolysis, improved bioenergetics and increased cell proliferation.

## Discussion

The endogenous production of H_2_S is highly upregulated in epithelial colorectal and prostate cancer cells, and in tumor-derived endothelial cells [28], [29]. In line with this evidence, we show that epithelial cancer cells (liver, breast, skin) innately produce large amounts of hydrogen sulfide independent of their origin. H_2_S is increased even further in cancer cells upon acute damage induced by hypoxia, hydrogen peroxide and bleomycin, or following recovery from damage as a result of increased expression of H_2_S-producing enzymes, *CBS* and *CTH*. These data are consistent with observations reported by others that *CTH* expression is upregulated by hypoxia leading to increased production of H_2_S [17].

H_2_S has been reported to have cytoprotective effects against hydrogen peroxide and doxorubicin induced toxicity and hypoxia [30]–[33]. We show here that DR cancer cells that recover from damage demonstrate cross tolerance to damaging conditions that induce death in a high number of parental control cancer cells. Moreover, when Pc cells are treated with NaHS, the cells develop resistance to damage. Together, our data suggest that an increase in H_2_S can occur in response to damage regardless of cell type or mode of damage. Based on such evidence, we suggest that increased H_2_S may be significant in the recovery of cancer cells from damages (e.g., oxidative stress, hypoxia, glucose deprivation, acidosis) that these cells endure in the harsh tumor microenvironments.

H_2_S is also essential to an increased proliferation in cancer cells recovered from damage. Consistent with our data, Cai *et al.* showed that treatment of HCT116 and SW480 colonic cancer cells with NaHS increased cell proliferation in these cancer cells [16]. The increase was dependent on Akt and ERK phosphorylation and blockade of Akt and ERK activation attenuated NaHS-induced cell proliferation. However contrary to such an observation, Jurkoska *et al.* showed that treatment of human neuroblastoma SH-SY5Y cells with NAC and ribose-cysteine, which results in elevation of hydrogen sulfide, leads to inhibition of cancer cell proliferation [34]. Furthermore, Cao *et al.* reported that treatment of WiDr colonic epithelial cancer cells with 50 to 200 µM NaHS for 24 hr suppresses viability [35]. One possible explanation for such paradoxical results may be attributed to sensitivity of certain cancer cells towards hydrogen sulfide mediated changes in the redox state. Another possibility is that this might be due to the fact that the effects of H_2_S are dose dependent. Thus, we show that H_2_S, in a dose-dependent manner, up-regulates Nampt, increases aerobic glycolysis and provides cytoprotection.

We show that in cancer cells that exhibit a high level of H_2_S, aerobic glycolysis and level of ATP and NAD^+^ are coordinately increased. We further show that such an increase in level of ATP and NAD^+^ in damage survivors is due to up-regulation of H_2_S production since forced increase in intracellular level of H_2_S leads to a concomitant rise in cellular pool of ATP and NAD^+^. In line with these observations, H_2_S is shown to improve mitochondrial ATP production following hypoxia [17]. Increased generation of H_2_S plays an important role in dictating cell survival after severe damage by promoting a reduction-oxidation balance, suppressing oxidative stress in mitochondria and increasing glutathione production [36].

We show that regardless of the type of injury, cancer cells that recover from damage show increased reliance on glycolysis as a main source of energy. Synthesis of ATP depends on the content of NAD^+^. Under glycolytic conditions, cells regenerate NAD^+^ via one of the recycling pathways; by either converting pyruvate into lactate or recycling nicotinamide (NAM) to NAD [37]. As shown here, the cyto-protective effects of H_2_S is related to its ability to increase cellular pool of ATP and NAD^+^ through a Nampt mediated response. Cancer cells, which are treated with H_2_S donor, NaHS, and cancer cells, which recover from damage, show an increased CBS and CTH driven H_2_S synthesis, and increased Nampt, the rate-limiting factor in NAD^+^ biosynthesis, and its product NAD^+^. Such changes should enable cells to survive when supplies of ATP and NAD^+^ are exhausted. Another possibility to consider is that, some, if not all of the increase in ATP in cancer cells, might be due to the direct utilization of H_2_S as a substrate in ATP generation by cancer cells. Recent evidence suggests that, in mammalian cells, H_2_S can serve as an electron donor and an inorganic source of energy. While low concentrations of H_2_S (0.1–1 µM) elicit a significant increase in mitochondrial function, its higher concentrations (3–30 µM) have an opposite effect on cellular bioenergetics [18]. Based on such data, it has been suggested that intra-mitochondrial H_2_S complements and balances the bioenergetic role of Krebs cycle-derived electron donors. Consistent with these observations, our data show that accumulation of H_2_S and upregulation of Nampt contribute to glycolytic activity, whereas glycolytic inhibitors were ineffective towards H_2_S and Nampt implying that H_2_S-Nampt is the leading cause of changes associated with adoption of glycolysis.

Our data show that FK866, a highly specific non-competitive inhibitor of Nampt, reduces expression of H_2_S-producing enzymes, diminishes production of H_2_S, and leads to depletion of ATP in cancer cells that recover from damage. Consistent with our data, it has been shown that FK866 which depletes the cellular pool of NAD^+^, induces tumor cell apoptosis while nicotinic acid and nicotinamide (Vitamin B3) oppose such effects of FK866 [38]. FK866 causes attenuation of glycolysis at the glyceraldehyde 3-phosphate dehydrogenase step which leads to restricted carbon flow from glycolysis resulting in reduced serine biosynthesis [27]. Therefore, FK866 mediated inhibition of H_2_S production might be attributed to reduced biosynthesis of serine and its utilization by CBS, which produces H_2_S and cystathionine. Cystathionine, in turn, is utilized by CTH to further produce H_2_S and cysteine. The existence of this positive feedback loop between H_2_S and Nampt may contribute to glycolytic activity and survival of cancer cells in face of microenvironmental challenges as well as after drug treatment. One of the downstream targets of Nampt is Sirtuin (Sirt1) that was reported to have impact on the survival mechanism [39]. Our preliminary data demonstrated that Sirtinol that acts as an inhibitor of Sirt1, did not affect the level of ATP in H_2_O_2_ treated control or DR cells (data not shown).

Consistent with the *in vitro* data, we show that damage recovered cancer cells derived from tumors generated *in vivo* exhibit a high level of H_2_S and Nampt and concomitantly show increased glycolysis, ATP and NAD^+^ production. According to Oncomine database, H_2_S-producing enzymes, CBS, CTH and MST as well as Nampt are all overexpressed in cancers of liver and breast and in melanomas (https://www.oncomine.org/) suggesting that this pathway operates in cancer cells of diverse origin. Together, our finding suggests that most cancers rely on H_2_S and Nampt to survive damages that they endure in their microenvironment.

In summary, a key attribute of cancer cells recovering from damage is accumulation of H_2_S that enables their recovery via a Nampt mediated metabolic change. H_2_S-Nampt pathway is a driving force in acquisition of a glycolytic phenotype, exponential growth and tolerance to damage. Therefore, even a small subset of tumor cells that recover from damage can give rise to generation of resistant cells that contribute to the expansion of cancer cell pool and facilitate their adaptation to a new microenvironment.

## Supporting Information

Figure S1 **Floating cells exhibit higher H_2_S level compare to bound cells.** HepG2 cells were treated with 800 µM H_2_O_2_ for 3 hr, then floating or bound cells were collected for H_2_S measurement. Mean values were compared to untreated control. ***; *p*<0.0005.
(TIF)

Figure S2 **Levels of H_2_S and** ***NAMPT*** **are not affected by glycolytic inhibitor.** The DR^H2O2 W2^ HepG2 cells were treated with HK1 inhibitor, 100 µM Bromopyruvic Acid, or LDH-A inhibitor, 1 mM Sodium Oxamate for 15 hr and levels of H_2_S (A) or Lactic Acid (B) were measured. Lactic Acid was measured by p-phenylphenol based colorimetric assay [40]. *; *p*<0.05, **; *p*<0.005. (C) *NAMPT* expression using Bromopyruvic Acid or Sodium Oxamate treated DR^H2O2 W2^ HepG2 cells. *β-ACTIN* was used as a loading control.
(TIF)

Figure S3 **NAD^+^ level decreases upon H_2_O_2_ damage.** HepG2 cells were treated with 800 µM H_2_O_2_ for 3 hr. NAD^+^ assay (A) was performed and Nampt expression (B) was measured by Western blotting. Mean values were normalized to total protein. β-actin served as a loading control. *; *p*<0.05.
(TIF)

Figure S4 **Level of NADH is increased in DR^H^ cells.** Level of NADH was measured in Pc and DR^H^ HepG2 cells by NAD^+^/NADH assay kit following manufacturer's instruction. Level of NADH was normalized to the protein content.
(TIF)

Figure S5 **Viability of cancer cells treated with FK866.** Pc and DR^H2O2 W2^ HepG2 cells were treated with 0, 1, 10, 100 and 200 nM with FK866 for 18 hr and performed XTT assay. Data are expressed as percentage (%) of control. There were no statistically significant changes in cell viability of cancer cells treated with any concentration of FK866.
(TIF)
