# Supplementary figures and images for: A H2S-Nampt Dependent Energetic Circuit Is Critical to Survival and Cytoprotection from Damage in Cancer Cells
Source: PLoS One. 2014 Sep 23;9(9):e108537. doi: 10.1371/journal.pone.0108537 (PMC4172766; doi:10.1371/journal.pone.0108537)

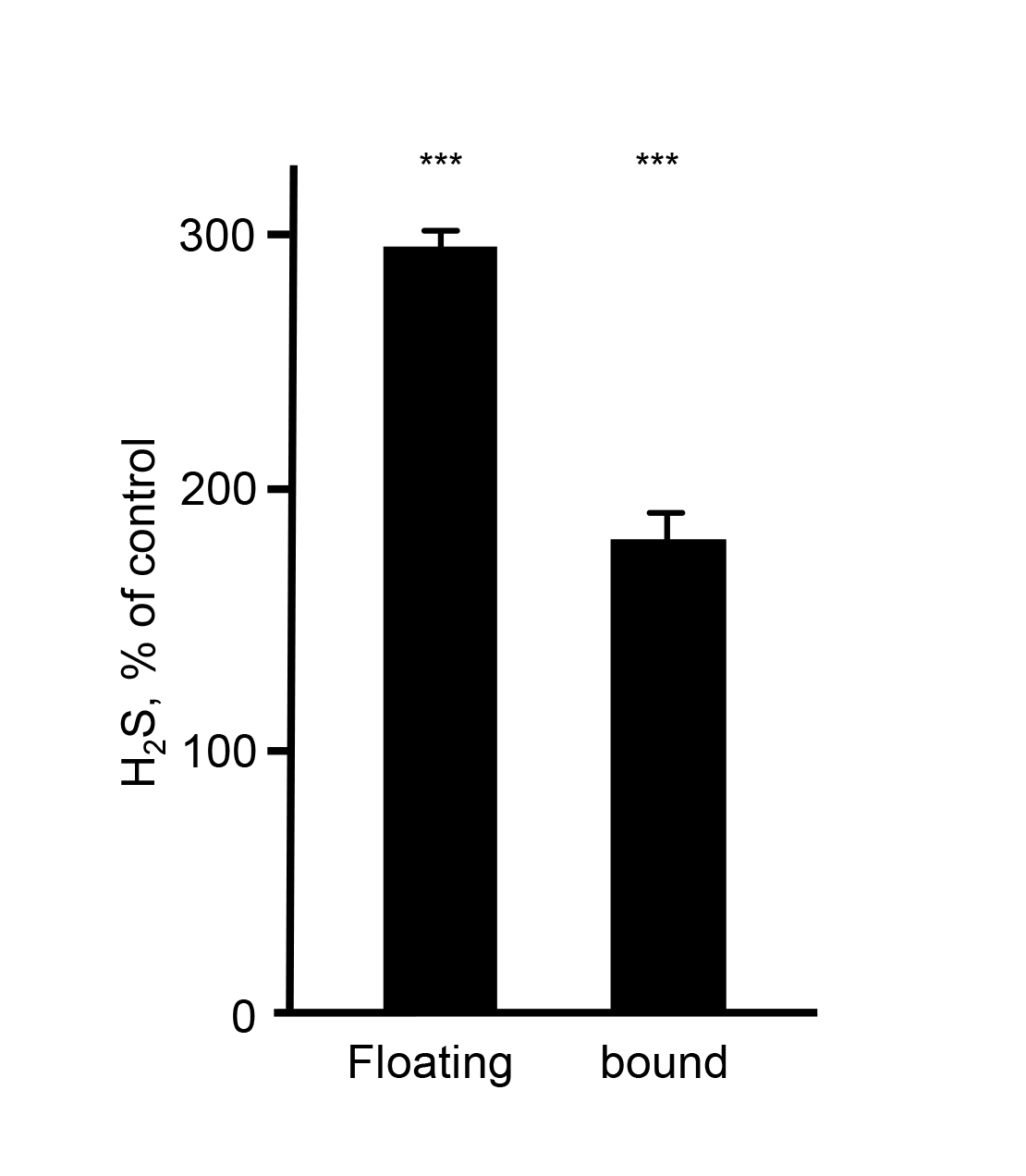

Supplement: Figure S1 — Floating cells exhibit higher H2S level compare to bound cells. HepG2 cells were treated with 800 µM H2O2 for 3 hr, then floating or bound cells were collected for H2S measurement. Mean values were compared to untreated control. ***; p<0.0005. (TIF) [file pone.0108537.s001.tif]

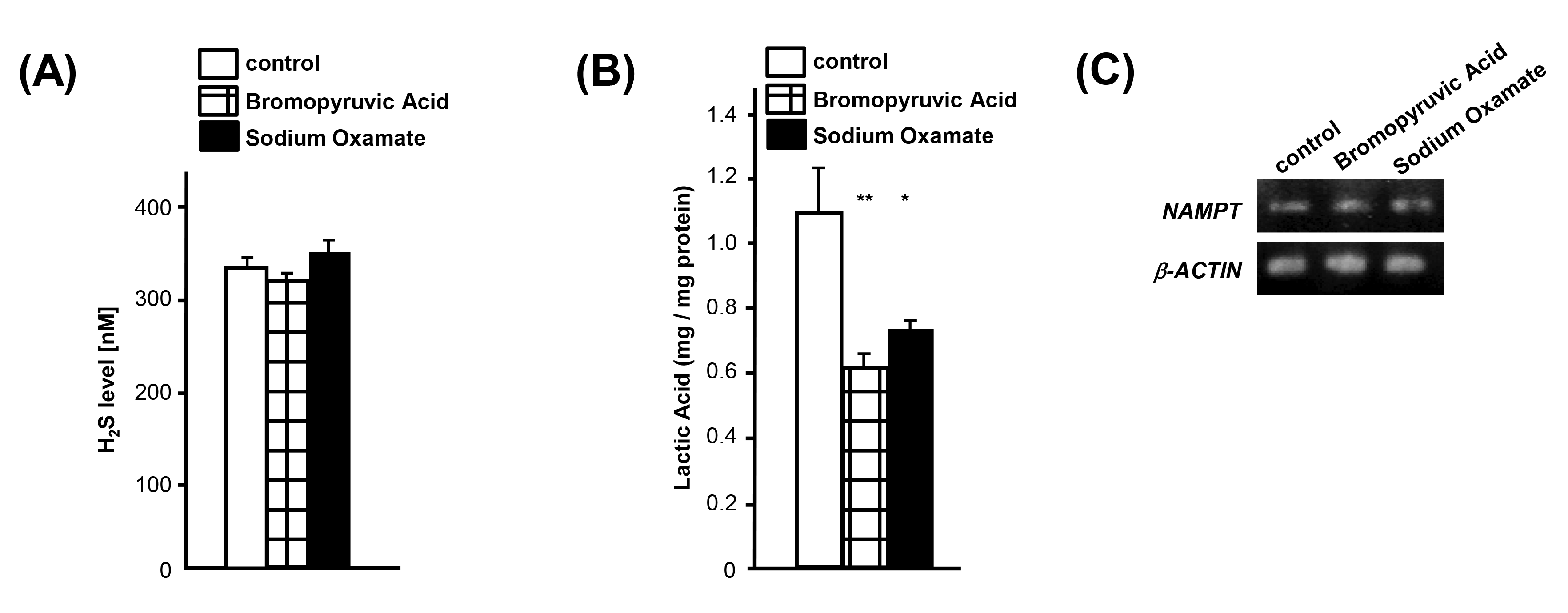

Supplement: Figure S2 — Levels of H2S and NAMPT are not affected by glycolytic inhibitor. The DRH2O2 W2 HepG2 cells were treated with HK1 inhibitor, 100 µM Bromopyruvic Acid, or LDH-A inhibitor, 1 mM Sodium Oxamate for 15 hr and levels of H2S (A) or Lactic Acid (B) were measured. Lactic Acid was measured by p-phenylphenol based colorimetric assay [40]. *; p<0.05, **; p<0.005. (C) NAMPT expression using Bromopyruvic Acid or Sodium Oxamate treated DRH2O2 W2 HepG2 cells. β-ACTIN was used as a loading control. (TIF) [file pone.0108537.s002.tif]

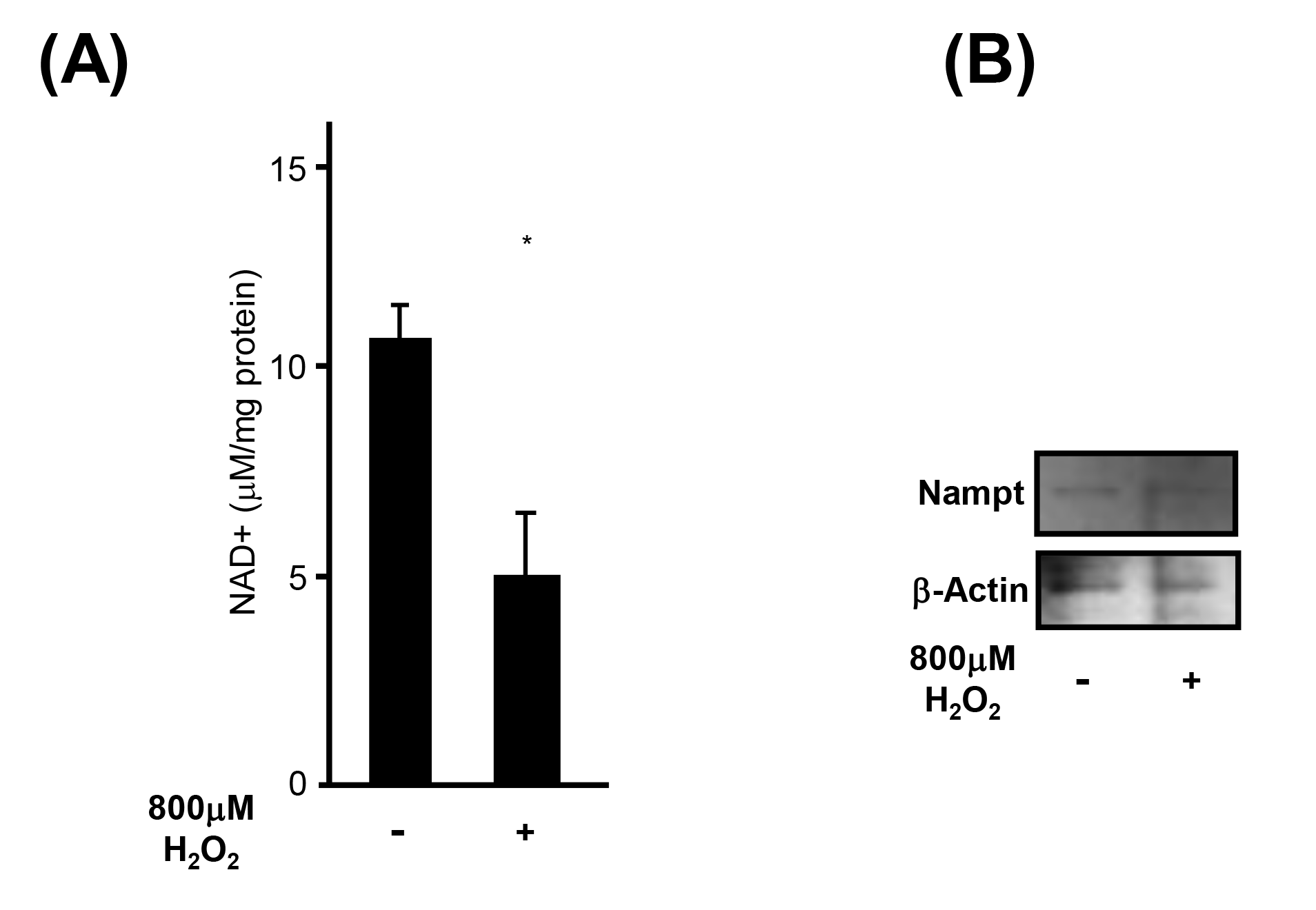

Supplement: Figure S3 — NAD+ level decreases upon H2O2 damage. HepG2 cells were treated with 800 µM H2O2 for 3 hr. NAD+ assay (A) was performed and Nampt expression (B) was measured by Western blotting. Mean values were normalized to total protein. β-actin served as a loading control. *; p<0.05. (TIF) [file pone.0108537.s003.tif]

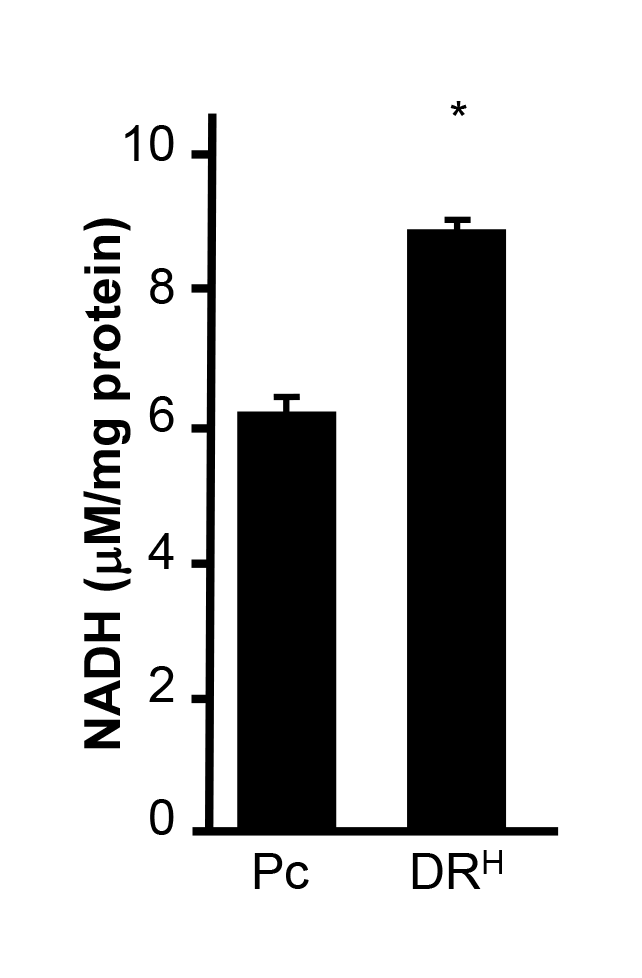

Supplement: Figure S4 — Level of NADH is increased in DRH cells. Level of NADH was measured in Pc and DRH HepG2 cells by NAD+/NADH assay kit following manufacturer's instruction. Level of NADH was normalized to the protein content. (TIF) [file pone.0108537.s004.tif]

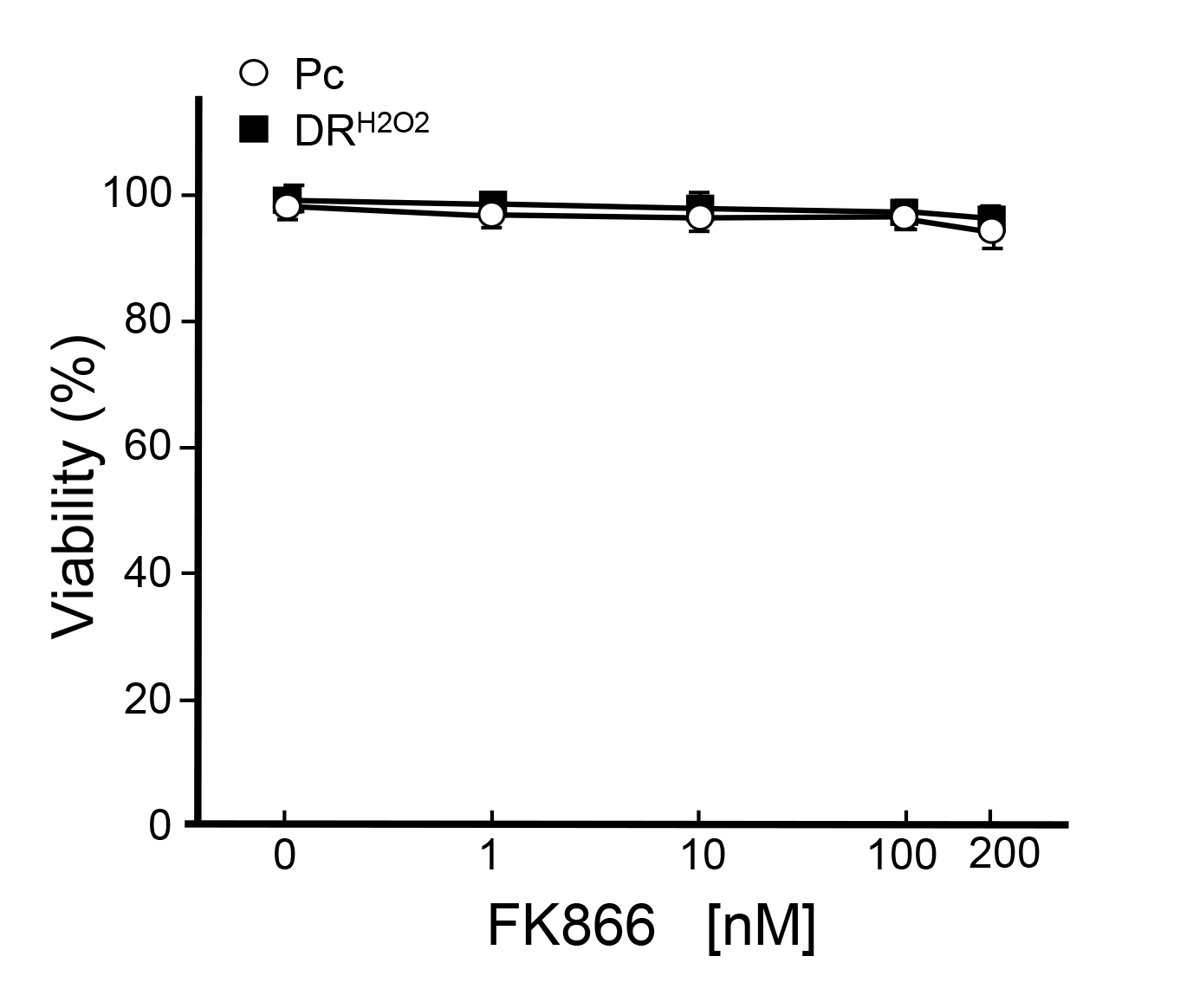

Supplement: Figure S5 — Viability of cancer cells treated with FK866. Pc and DRH2O2 W2 HepG2 cells were treated with 0, 1, 10, 100 and 200 nM with FK866 for 18 hr and performed XTT assay. Data are expressed as percentage (%) of control. There were no statistically significant changes in cell viability of cancer cells treated with any concentration of FK866. (TIF) [file pone.0108537.s005.tif]
